# Improvements in advanced hepatocellular carcinoma to repeat implementation of primary protocol after cancer progression occurs following sequential systemic therapy and a clinical trial: A case report

**DOI:** 10.1097/MD.0000000000038138

**Published:** 2024-05-10

**Authors:** Hongwei Huang, Qiaoqiao Wei, Chao Leng, Hao Wang, Bin Mei

**Affiliations:** a Hepatic Surgery Center, Tongji Hospital, Tongji Medical College, Huazhong University of Science and Technology, Wuhan, China.

**Keywords:** hepatocellular carcinoma, subsequent therapy, systemic therapy, transcatheter arterial chemoembolization

## Abstract

**Introduction:**

Systemic therapy is recommended for patients with advanced hepatocellular carcinoma (aHCC). However, drug resistance occurs over time when patients receive systemic therapy, resulting in cancer progression. Due to the lack of relevant clinical trials, optimizing subsequent treatments after cancer progression remains elusive.

**Patient concerns:**

A 52-year-old male patient presented with epigastric discomfort and fatigue for almost 1 month with a past history of chronic hepatitis B virus infection for 30 years.

**Diagnosis:**

Based on the patient’s performance status, tumor status assessed by computed tomography, liver function, he was diagnosed with HCC at BCLC stage C.

**Interventions and Outcomes:**

He first received transarterial chemoembolization (TACE) combined with sintilimab and lenvatinib as first-line treatment and experienced 10-month progression-free survival. After cancer progression, the patient participated in a clinical trial of ABSK-011, a novel fibroblast growth factor receptor 4 inhibitor, with a frustrating result. Then, the patient underwent TACE and received sintilimab plus lenvatinib again. Surprisingly, the tumor had a partial response, and the patient’s serum alpha-fetoprotein returned to normal.

**Lessons:**

The combined treatment of TACE plus systemic therapy might be an appropriate subsequent treatment.

## 1. Introduction

Liver cancer is a common malignancy worldwide that ranks sixth in incidence and third in mortality globally among all cancers, with hepatocellular carcinoma (HCC) accounting for approximately 80% of all liver cancer cases.^[1]^ More than 70% of HCC patients are diagnosed at an advanced stage, which precludes curative surgery.^[2]^ According to the guidelines of the American Society of Clinical Oncology (ASCO), systemic therapy, including atezolizumab plus bevacizumab and tyrosine kinase inhibitors (TKIs), such as lenvatinib and sorafenib, is recommended as the first-line treatment for advanced HCC (aHCC).^[3]^ Additionally, combined therapy of locoregional treatment plus systemic therapy to further improve outcomes is an active area of research. Several studies demonstrate that transarterial chemoembolization (TACE), a kind of locoregional treatment, in combination with TKIs and immune checkpoint inhibitors (ICIs) may improve survival outcomes with tolerable toxicity for patients with aHCC.^[4]^

Notably, many patients experience drug resistance and cancer progression when they receive systemic therapy for a period and, thus, require alternative types of effective treatment. According to the ASCO guidelines, second-line therapies for aHCC patients include other TKIs (regorafenib or cabozantinib), ramucirumab and ICIs (pembrolizumab or nivolumab).^[3]^ Few studies have discussed appropriate subsequent treatments for HCC patients with cancer progression after resistance to combined therapy. A retrospective study showed that HCC patients with cancer progression after receiving combined therapy could achieve survival benefits from subsequent programmed death-1 (PD-1) inhibitors plus locoregional therapy, and this was observed particularly among patients who were initially resistant to PD-1 inhibitors.^[5]^ In addition, some studies showed that subsequent single-agent TKIs or in combination with programmed death ligand-1 (PD-L1)/cytotoxic T lymphocyte-associated antigen-4 (CTLA-4) inhibitors show promising results with an approximately 15% response rate for patients who received prior ICI-based therapies.^[6]^ Further studies on the optimal subsequent treatment are greatly needed.

Here, we report a patient with aHCC who received TACE plus TKIs and ICIs as first-line treatment. At first, the tumor achieved a partial response (PR) with this protocol but then progressed in the 16th month. The patient then participated in a clinical trial of a novel drug called ABSK-011, a fibroblast growth factor receptor 4 (FGFR4) small molecule inhibitor. The patient experienced progressive disease (PD) and dropped out of the trial after using the drug for 3 cycles over a period of almost 2 months. Finally, the patient returned to the primary protocol owing to economic factors. The tumor achieved PR again and the alpha-fetoprotein level returned to normal after switching to the primary protocol for 3 months. To our knowledge, this phenomenon has not been previously reported. This study was approved by the Ethics Committee of Tongji Hospital affiliated to Huazhong University of Science and Technology, and written informed consent was obtained from the patient.

## 2. Case presentation

**Figure 1.**
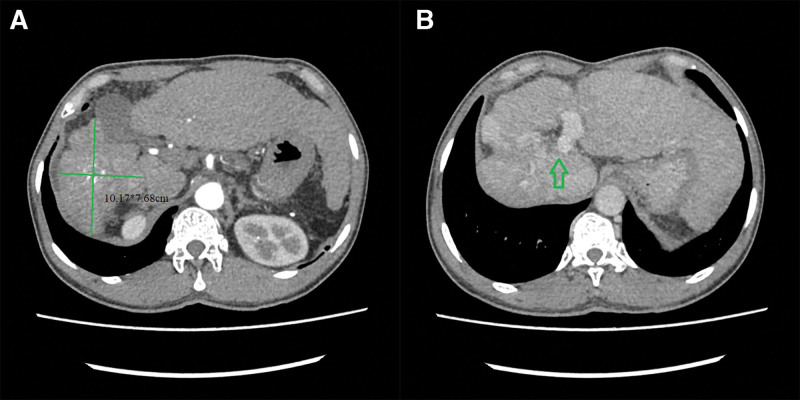
(A) The largest cross-sectional area of the tumor (10.17*7.68 cm). (B) The green arrow indicates tumor thrombus in the right branch of the portal vein.

In July 2020, a 52-year-old man presented with epigastric discomfort and fatigue for almost 1 month. He complained that he always felt distension in his right upper quadrant abdominal without vomiting. He had a history of chronic hepatitis B virus infection for almost 30 years. He underwent splenectomy because of hypersplenism 10 years ago. On physical examination, he was found to have mild tenderness in the right upper quadrant of the abdomen without guarding, rigidity or rebound tenderness. Laboratory results showed a total bilirubin level of 18.1 µmol/L; an albumin level of 40.1 g/L; alanine aminotransferase level of 21 U/L; aspartate aminotransferase level of 44 U/L; prothrombin time of 14.2 seconds; and unusually high levels of serum alpha-fetoprotein (AFP) (49,109 ng/mL) and protein induced by vitamin K absence or antagonist-II (7967 mAu/mL). Computed tomography (CT) imaging indicated a target lesion located in the right hepatic lobe, measuring up to 10.17*7.68 cm, with the characteristic radiological appearance of HCC (hyperenhancement during the arterial phase and washout in the venous phase), and compensatory hyperplasia in the left lobe combined with a tumor thrombus in the right branch of the portal vein (Fig. 1). The gastroscope showed severe gastroesophageal varices with a red color sign. The patient had no ascites or hepatic encephalopathy. The Child–Pugh score was 5 (level A). Based on the performance status (Eastern Cooperative Oncology Group score, 1), tumor status, and liver function, the patient was diagnosed with aHCC according to the ASCO guidelines.^[3]^

**Figure 2.**
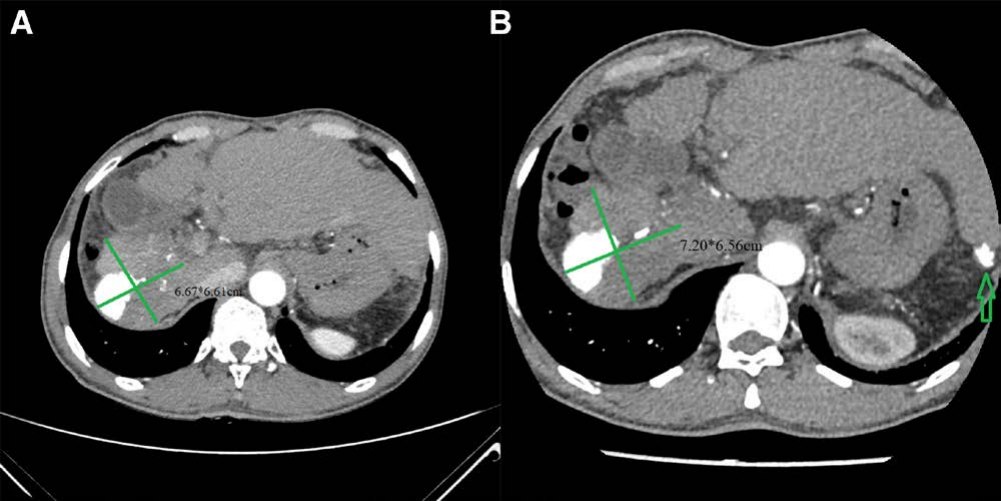
(A) The target lesion shrunk to 6.67*6.6 cm. (B) The target lesion grew to 7.20*6.56 cm; the green arrow shows a new lesion with iodinated oil deposition in the left hepatic lobe.

The patient underwent TACE combined with sintilimab (a kind of PD-1 inhibitor, 200 mg intravenous injection once every 3 weeks) and lenvatinib (8 mg orally per day). The patient underwent TACE procedure 4 times, which was repeated for 4 to 6 weeks. The first use of sintilimab and lenvatinib was in November 2020. During the first 10 months, abdominal CT showed that the target lesion shrunk to 6.61*6.66 cm (Fig. 2A) with decreased levels of AFP and protein induced by vitamin K absence or antagonist-II down to 1823.00 ng/mL and 118 mAu/mL, respectively. The bone scan and chest CT showed that there was no extrahepatic metastasis. The cancer achieved PR according to modified Response Evaluation Criteria in Solid Tumors criteria. In the thirteenth month, he received TACE again, considering that the level of AFP rose up to 9327 ng/mL. CT imaging performed in the sixteenth month presented a new lesion with iodinated oil deposition in the left hepatic lobe, and the right lesion grew to 7.20*6.56 cm (Fig. 2B). The level of serum AFP was 7894 ng/mL. The cancer appeared to have progressed.

**Figure 3.**
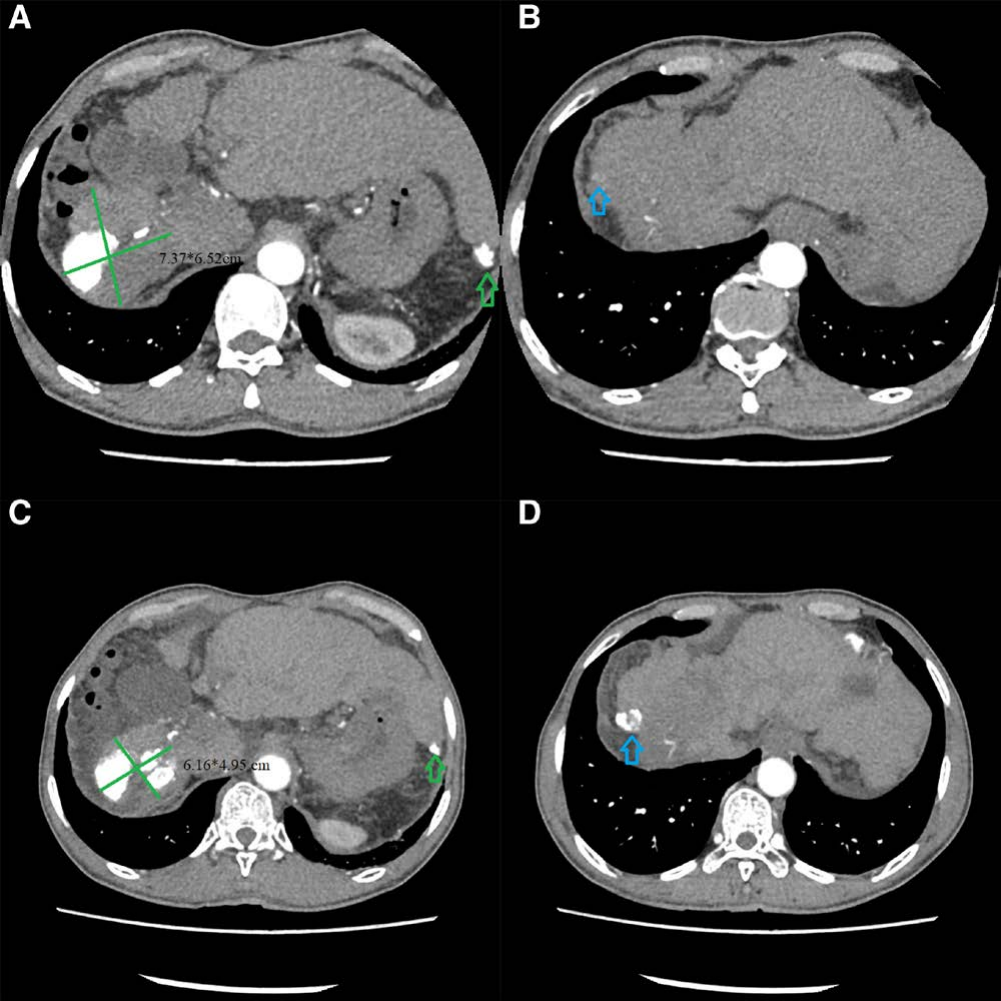
(A) The target lesion grew to 7.37*6.52 cm with no obvious change in the left lobe lesion (the green arrow). (B) The blue arrow shows a new enhancement nidus in Couinaud segment 8. (C) The target lesion shrunk to 6.16*4.95 cm with no obvious change in the left lobe lesion (indicated by the green arrow). (D) The blue arrow shows iodinated oil deposition in the lesion of segment 8.

The patient continued to receive sintilimab and lenvatinib for 3 months, but the AFP level continued to rise to 14,105 ng/mL. Then, he chose to try alternative therapy and participated in a clinical trial of a new drug called ABSK-011, which is an FGFR4 small molecule inhibitor developed by Shanghai Heyu Biomedical Technology Company. The patient’s dosing regimen was 180 mg of ABSK-011 orally per day plus intravenous infusion of 1200 mg atezolizumab once every 3 weeks. Atezolizumab, which is different from sintilimab, is a kind of PD-L1 inhibitor. After 3 cycles of treatment for almost 2 months, the AFP levels increased to 27,080 ng/mL, and the cancer was evaluated as PD because an abdominal CT showed that the target lesion in the right hepatic lobe measured 7.37*6.52 cm, there was a new lesion (1.52*1.07 cm) in Couinaud segment 8, and there was no apparent change in the lesion of left hepatic lobe (Fig. 3A and B).

Then, the patient decided to drop out of the clinical trial and underwent TACE again on March 23, 2022. Owing to the donation policy of original drugs, he received lenvatinib plus sintilimab on May 6, 2022. Fortunately, it was discovered that the levels of tumor markers returned to normal levels 3 months later. The abdominal CT showed that the target lesions appeared to be shrink to 6.16*4.95 cm, while there was iodinated oil deposition in the lesion of segment 8 and no obvious change in the lesion of the left hepatic lobe (Fig. 3C and D). The tumor achieved PR again. No disease progression occurred during the follow-up until June 12, 2023.

**Figure 4.**
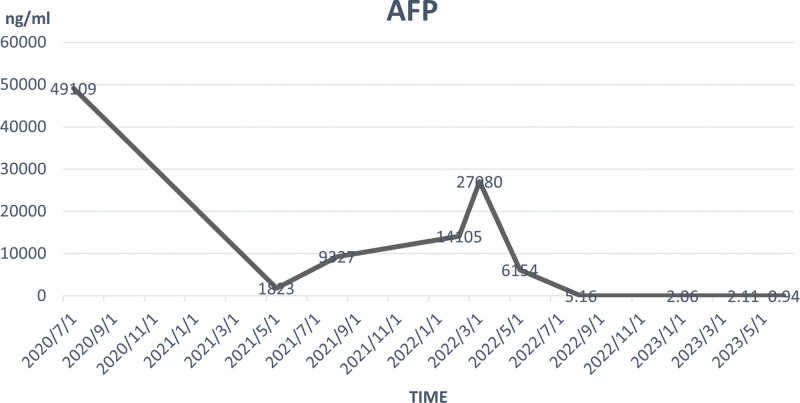
Variations in alpha-fetoprotein levels.

Throughout the treatment period, the patient experienced no obvious discomfort or adverse events related to antiangiogenic drugs or immunotherapy. The hepatitis B virus replication level was well controlled. The variations in serum AFP levels throughout the whole course are illustrated in Figure 4.

## 3. Discussion

Here, we present a patient with aHCC who underwent antitumor synthetic treatment for almost 3 years. In the first stage, the tumor achieved PR through TACE combined with sintilimab and lenvatinib for almost 1 year. In the second stage, the patient participated in a clinical trial which resulted in cancer progression. In the third stage, the cancer achieved PR again when he switched to the primary protocol. This is a rare phenomenon that has not yet been reported. We would like to report this case to stimulate discussions on appropriate forms of subsequent treatment.

Recently, combined antiangiogenic therapy plus immunotherapy has become the focus of scientific and clinical research and has shown promising results. Based on the results of the IMbrave150 trial, the combination of atezolizumab with bevacizumab was recommended as the first-line therapy for most patients with aHCC.^[3]^ Sintilimab combined with antiangiogenic therapy could improve survival outcomes and was approved in the first-line setting for aHCC patients.^[7]^ The patient refused to receive atezolizumab plus bevacizumab owing to the high risks of upper gastrointestinal hemorrhage and the high cost. Therefore, sintilimab plus lenvatinib might be an appropriate combination for our case.

Performing TACE in aHCC patients remains controversial. Some experts view TACE as unsuitable for patients with aHCC considering the risk of complications after the procedure, such as liver failure and intrahepatic tumor progression.^[8]^ Nevertheless, other indicated that TACE could be appropriate for aHCC patients with vascular invasion and adequate liver function. The results of the LAUNCH trial successfully demonstrated the validity of the combination of TACE and lenvatinib as the first-line therapy for patients with aHCC.^[9]^ Moreover, some studies reported that the triple combination of TACE plus lenvatinib and PD-1 inhibitors yield a better median overall survival (16.9–23.6 months), median progression-free survival (7.3–13.3 months) and objective response rate (46.7%–56.1%) with tolerable toxicity.^[4,10]^ In our case, the tumor achieved PR, and progression-free survival was 10 months without any adverse events. These results indicate that the triple therapy of TACE combined with TKIs plus ICIs might be a superior treatment option for aHCC patients. The mechanism underlying this phenomenon might be explained as follows: TACE effectively causes tumor necrosis and reduce tumor burden, resulting in the release of tumor-specific antigens, which consequently improve the effects of immunotherapy.^[11]^ Furthermore, TKIs may offset post-TACE hypoxia-induced angiogenesis to inhibit tumor growth.^[9]^ Antiangiogenic effects of TKIs can synergistically enhance the antitumor effects of ICIs by modulating the tumor microenvironment (TME). For instance, TKIs can reduce the secretion of immunosuppressive cytokines such as TGF-β and IL-10 and inhibit immunosuppressive regulatory T cells, macrophages and myeloid-derived suppressor cells.^[12]^ TKIs promote tumor vascular normalization and consequently improve the delivery of embolism agents and systemic anticancer drugs to optimize the anticancer effects of TACE and immunotherapy.^[13]^ Many other studies are currently underway to validate the efficacy and safety of various combination therapies.

Unfortunately, most patients do not experience long-term benefits, largely because they develop drug resistance. The mechanisms of drug resistance are obscure and have not been thoroughly illustrated until now. It is well known that the TME plays a virtual role in tumor initiation, invasiveness, and resistance. Various receptor tyrosine kinases constitute a complex network of signal transduction pathways in cells. Even though some signaling pathways are inhibited in tumor cells, other pathways can still transduce the signal and may be upregulated compensatorily resulting in drug resistance. For example, high levels of EGFR were associated with resistance to lenvatinib in HCC patients because the inhibition of FGFR by lenvatinib led to aberrant activation of the EGFR/PAK2/ERK5 signaling axis.^[14]^ Further studies are needed to illustrate the mechanisms of drug resistance and overcome it.

It can be inferred that the number of patients who experience HCC progression after first-line therapy will increase significantly. Improving postprogression survival has become an urgent need for these patients. However, it remains elusive what would be the optimal subsequent therapy for patients who experience disease progression after combined treatment of locoregional therapy with TKIs and ICIs. In our case, the tumor responded to the subsequent treatment, which inspired us. First, the TACE treatment that he received might successfully release tumor neoantigens as obvious targets for the immune system. A retrospective study showed that patients with HCC progression after combined treatment could benefit from subsequent PD-1 inhibitors plus locoregional therapy.^[5]^ This emphasizes the necessity for locoregional therapy in patients with good liver function. Second, atezolizumab, a monoclonal PD-L1 inhibitor, could directly bind to PD-L1 on tumor cells and block its interaction with PD-1 expressed on T cells and antigen-presenting cells. PD-L1 inhibitors combined with CTLA-4 inhibitors or other TKIs might be options worth considering for prolonging survival in patients with drug resistance. In addition, ABSK-011, an FGFR4 small molecule inhibitor, may ameliorate the crosstalk between receptor tyrosine kinases and promote tumor vascular normalization. The TME might be altered by ABSK-011 and atezolizumab. Notably, the findings of post hoc analyses of the REFLECT study illustrated that patients can experience survival benefits from subsequent TKIs.^[15]^ Therefore, other TKIs, such as regorafenib, might be effective after progression occurs in patients taking lenvatinib plus PD-1 inhibitors.

## 4. Conclusion

Triple therapy of TACE combined with lenvatinib and sintilimab was effective as a first-line therapy for patients with aHCC. Subsequent therapy of TACE combined with other TKIs and/or PD-L1/CTLA-4 inhibitors might improve patient survival. Further investigations are needed to understand how patients overcome drug resistance and to determine effective subsequent therapies for patients with HCC.

## Acknowledgment

We thank the patient in this report and his family.

## Author contributions

**Conceptualization:** Bin Mei.

**Data curation:** Hongwei Huang, Qiaoqiao Wei, Chao Leng, Hao Wang.

**Formal analysis:** Hongwei Huang, Qiaoqiao Wei, Hao Wang.

**Investigation:** Hongwei Huang, Qiaoqiao Wei, Chao Leng, Hao Wang.

**Methodology:** Chao Leng, Bin Mei.

**Project administration:** Bin Mei.

**Supervision:** Chao Leng, Bin Mei.

**Validation:** Bin Mei.

**Writing – original draft:** Hongwei Huang.

**Writing – review & editing:** Chao Leng, Bin Mei.
